# Radiofrequency localization of nonpalpable breast cancer in a multicentre prospective cohort study: feasibility, clinical acceptability, and safety

**DOI:** 10.1007/s10549-023-07006-x

**Published:** 2023-06-15

**Authors:** Anke Christenhusz, Bianca M. den Dekker, Thijs van Dalen, Lisa Jongen, Margreet C. van der Schaaf, Lejla Alic, Bennie ten Haken, Ruud M. Pijnappel, Anneriet E. Dassen

**Affiliations:** 1 Department of Surgery, Medisch Spectrum Twente Enschede, Enschede, The Netherlands; 2 Department of Radiology, University Medical Center Utrecht, Utrecht University, Utrecht, The Netherlands; 3 Department of Surgery, Diakonessenhuis Utrecht, Utrecht, The Netherlands; 4 Department of Radiology, Diakonessenhuis Utrecht, Utrecht, The Netherlands; 5 Department of Radiology, Medisch Spectrum Twente Enschede, Enschede, The Netherlands; 6 Dutch Expert Centre for Screening, Nijmegen, The Netherlands; 7 University of Twente, Magnetic Detection and Imaging Group, Enschede, The Netherlands

**Keywords:** Breast cancer, Primary lesion localization, Breast conserving surgery, Radiofrequency identification, RFID

## Abstract

**Purpose:**

In breast conserving surgery, accurate lesion localization is essential for obtaining adequate surgical margins. Preoperative wire localization (WL) and radioactive seed localization (RSL) are widely accepted methods to guide surgical excision of nonpalpable breast lesions but are limited by logistical challenges, migration issues, and legislative complexities. Radiofrequency identification (RFID) technology may offer a viable alternative. The purpose of this study was to evaluate the feasibility, clinical acceptability, and safety of RFID surgical guidance for localization of nonpalpable breast cancer.

**Methods:**

In a prospective multicentre cohort study, the first 100 RFID localization procedures were included. The primary outcome was the percentage of clear resection margins and re-excision rate. Secondary outcomes included procedure details, user experience, learningcurve, and adverse events.

**Results:**

Between April 2019 and May 2021, 100 women underwent RFID guided breast conserving surgery. Clear resection margins were obtained in 89 out of 96 included patients (92.7%), re-excision was indicated in three patients (3.1%). Radiologists reported difficulties with the placement of the RFID tag, partially related to the relatively large needle-applicator (12-gauge). This led to the premature termination of the study in the hospital using RSL as regular care. The radiologist experience was improved after a manufacturer modification of the needle-applicator. Surgical localization involved a low learning curve. Adverse events (*n* = 33) included dislocation of the marker during insertion (8%) and hematomas (9%). The majority of adverse events (85%) occurred using the first-generation needle-applicator.

**Conclusion:**

RFID technology is a potential alternative for non-radioactive and non-wire localization of nonpalpable breast lesions.

**Supplementary Information:**

The online version contains supplementary material available at 10.1007/s10549-023-07006-x.

## Introduction

The detection of nonpalpable breast cancer has increased by improved imaging and the introduction of population-based screening programmes [1]. Significantly more patients opt for breast conserving surgery (BCS) in combination with radiotherapy, as it is a proven alternative to mastectomy with respect to overall survival [2]. In BCS, accurate intraoperative lesion localization is essential for adequate surgical margins whilst sparing surrounding healthy tissue to achieve optimal cosmesis [3]. Preoperative wire localization and radioactive seed localization are widely accepted methods to guide surgical excision of nonpalpable breast lesions but have significant limitations [4].

Wire-guided localization (WL) has been the standard method for the surgical excision of nonpalpable tumours for several decades. WL is facilitated by an imaging guided insertion of a wire in the tumour by a radiologist. This technique requires careful logistical planning as the wire needs to be placed on the day of surgery, which can lead to significant workflow inefficiencies and delays. In addition, the entry site of the wire may differ from the ideal surgical approach. Other disadvantages of WL are patient anxiety caused by the wire protruding from the patient’s skin, the risk of migration, dislodgement or pneumothorax during placement [5]. Therefore, radioactive seed localization (RSL) has been introduced to improve the surgical treatment. During a short procedure, a radioactive seed (^125^I) is placed in the tumour in a short radiological procedure which subsequently is localised intraoperatively by a gamma probe. The RSL resection margins, re-excision, and reoperation rates are non-inferior to WL. However, the use of radioactive seeds is constrained by stringent nuclear regulations needing meticulous tracking of the radioactive seed legislation on top of a growing concern for availability of radioactive materials for medical use [6]. These disadvantages have limited the widespread adoption of the radioactive seed and have prompted research and development of non-radioactive seed.

Recently introduced radiofrequency identification (RFID) technology may offer a viable non-radioactive, non-wire alternative for localization of nonpalpable breast lesions during BCS. A number of single centre cohort studies [7–11] and a retrospective cohort analysis [12] have concluded that the RFID lesion localization can be used safely and effectively for non-palpable breast lesions. However, data to evaluate RFID localization process is currently lacking. Therefore, the current research evaluates the feasibility, clinical acceptability and safety of RFID surgical guidance for localization of nonpalpable breast cancer in a prospective multicentre cohort study.

## Materials and methods

The design of this prospective multicentre cohort study, and technical specifications about the RFID technique were reported in detail earlier [13] (The Dutch Trial Register—NL8019) concluding an unattainable response assessment of neoadjuvant chemotherapy (NAC) for the patients with a RFID tag due to a significant MRI artefact. The study was approved by the Medical Research Ethical Committee of the University Medical Center Utrecht. Informed consent was obtained from all participants.

### Study population

All adult breast cancer patients with nonpalpable, histologically proven, ductal carcinoma in situ or invasive breast cancer, scheduled for breast conserving surgery between April 2019 and May 2021 in Medisch Spectrum Twente (Enschede, the Netherlands) or Diakonessenhuis Utrecht (Utrecht, the Netherlands), were considered eligible for participation. A total of 100 patients were included in this feasibility study. Participants were recruited at the surgical outpatient clinic. The following exclusion criteria were applied: lesion located deeper than 7 cm assessed in supine position, pregnancy or lactating status, and multicentric breast cancer. Additional exclusion criterion was applied with respect to the standard of care for localization at each study site:

- At Diakonessenhuis Utrecht (RSL) patients undergoing neoadjuvant chemotherapy treatment (NAC) were excluded, as a single procedure using RSL was preferred over a two-step localization procedure with a biopsy marker before NAC followed by preoperative RFID tag placement [13].
- At Medisch Spectrum Twente (WL) patients undergoing NAC were not excluded. A biopsy marker was used during NAC, followed by preoperative RFID tag placement.

### RFID tag placement procedure

**Fig. 1:**
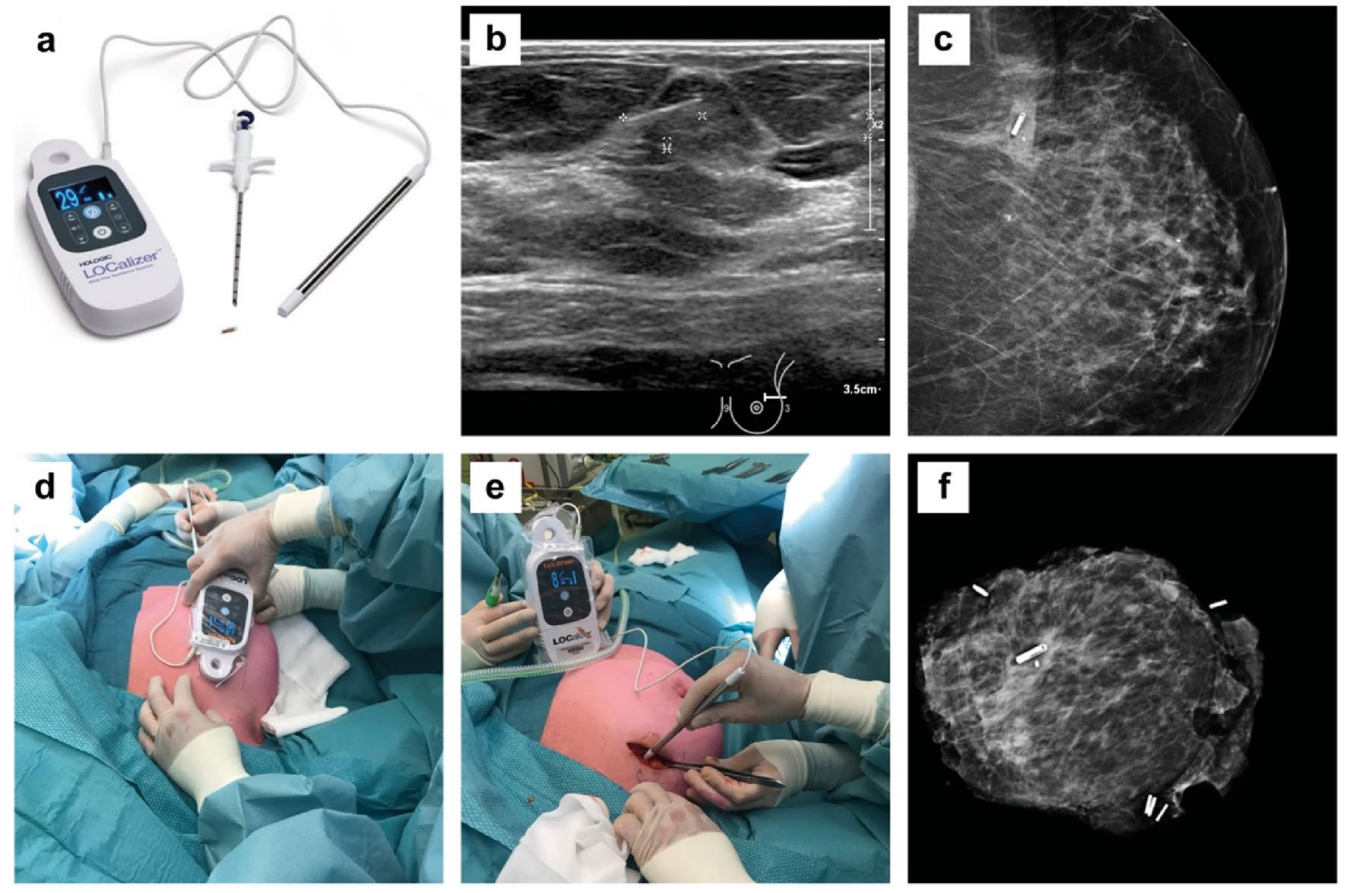
An overview of RFID localization procedure with **A** the RFID LOCalizer™ system (Faxitron, Hologic); **B** Ultrasound guided RFID tag placement; **C** Post placement mammography to confirm correct tag location (CC-view); **D** Transcutaneous detection with the loop probe; **E** Intraoperative detection with the surgical probe; **F** Specimen radiography (Trident, Hologic) showing the RFID tag, biopsy marker and specimen clips

The LOCalizer™ RFID tag (10.6 × 2 mm) (Fig. 1A) was inserted during an image guided procedure up to 30 days prior to surgery by an experienced breast radiologist or radiology resident under supervision (intended use > 30 days) [14]. The image guided procedure involved ultrasound (Fig. 1B) or stereotactic imaging. The RFID tag was inserted percutaneously through a small skin incision with a preloaded 12-gauge sterile needle-applicator after injection of local anaesthesia (lidocaine 2%). Each tag has a unique identification number and can be localised transcutaneous by a handheld reader making it easy to distinguish two different tags. The reader (illustrated in Fig. 1A) has a loop probe with a detection range of up to 7 cm, and an attachable single-use sterile pencil probe (8 mm tip diameter) with a detection range up to 3.5 cm for highly accurate localization. The audible and visual feedback provide the surgeon with real-time guidance during the excision procedure. During the course of this study, the manufacturer made a modification to the applicator needle allowing a comparison between the versions.

**Fig. 2:**
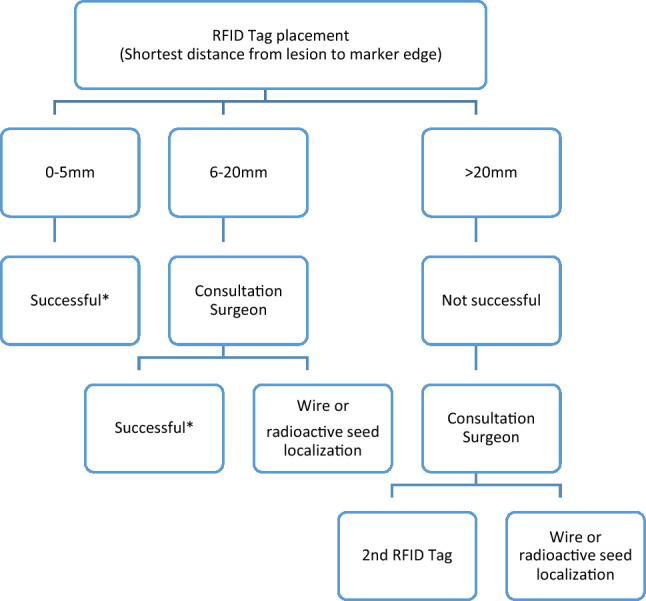
Decision tree defining a successful RFID procedure. *Successful placement was defined as 0–5 mm distance between any point of the tag to any point of the tumour. When the tag was 6–20 mm from the tumour the radiologist consulted the surgeon if the localization was acceptable

The quality of RFID placement was assessed by post-placement mammography and classified as successful if inserted at 0–5 mm omnidirectional distance to the tumours or the pre-NAC placed biopsy marker. In case of unsuccessful placement an additional marker was used, i.e. WL or radioactive seed in cases when an additional marker was needed at a distance shorter than 2 cm (to prevent possible signal interference [14]), or an RFID tag. For cases with a complete radiological response after NAC, the RFID tag was inserted next to the biopsy marker placed in the tumour before the start of NAC. The complete decision three is illustrated in Fig. 2. The pain score after local anaesthesia was recorded by the Visual Analogue Scale (VAS) [15]. Directly after the procedure the radiologist completed a questionnaire assessing the procedure and system usability [13, 16]. The total duration of the procedure was recorded (from start of local anaesthesia to the deposition of the marker). Directly after the tag placement, a two-view mammography, i.e. craniocaudal (CC) and mediolateral oblique (MLO) was acquired to assess the position of the RFID tag (Fig. 1C). The shortest distance from RFID tag to lesion (or biopsy clip marker) was recorded.

### RFID guided breast conserving surgery

All surgical procedures were performed by experienced breast cancer surgeons or surgery residents under direct supervision. The RFID tag was intraoperatively localised using the handheld portable reader device (Fig. 1A). Transcutaneous measurements were performed with the loop probe (Fig. 1D), measurements after incision were performed with a single-use sterile pencil probe (Fig. 1E). In addition to measurements with the portable reader device lumpectomy specimen radiography was performed to confirm successful retrieval of the RFID tag (Fig. 1F). Directly after the procedure the surgeon completed a questionnaire on the ease of the procedure and system usability [13] [16]. Duration of the procedure from primary incision until specimen retrieval was recorded. Postoperative complications were recorded up to 30 days postoperatively.

### Pathology

Lumpectomy specimens were examined by experienced breast pathologists to record the following parameters: presence and location of the RFID tag, resection margins, specimen dimensions, and tumour dimensions. The respective volumes were calculated using the three radii (1/2 of the diameter assessed by pathology protocol), and the ellipsoid equation $V=\frac{4}{3}\pi {r}_{1}{r}_{2}{r}_{3}.$ According to the Dutch breast cancer guidelines[17], surgical margins were classified as radical (no tumour in ink), focally irradical (tumour in 1 limited area ≤ 4 mm) and more than focal irradical [17]. Re-excision or mastectomy was recommended for patients with more than focal irradical margins, whilst in patients with focal irradical margins a radiation-boost will suffice.

### Data analysis

All data was collected using Castor Electronic Data Capture. The radical (re)excision rate is expressed as a quotient between the total number of excisions and the total number of radical (re)excisions. Descriptive statistics were used to summarise the data by mean, median, interquartile range (IQR), and 95% confidence intervals (CI). Data was analysed using IBM SPSS statistics (IBM Corp., Armonk, NY, USA, version 28).

## Results

Between April 2019 and May 2021, a total of 100 women (median age of 62, 54–69 IQR) underwent breast conserving surgery using RFID localization (Table 1): 91 procedures were performed at Medisch Spectrum Twente, and nine at the Diakonessen hospital Utrecht.

**Table 1:** Patient characteristics with Age, BMI, and cup-size collected from the patient record

Age, median (IQR*)	62 (54–69)
BMI, median (IQR)	26 (23–29)
Cup size (%)	
AA-A	2 (2%)
B	21 (21.4%)
C	29 (29.6%)
D	21 (21.4%)
E	16 (16.3%)
F-G-H	9 (9.2%)
Missing	2
Breast Density (%)	
A (< 25%)	17 (19.1%)
B (25–50%)	48 (53.9%)
C (50–75%)	21 (23.6%)
D (> 75%)	3 (3.4%)
Missing	11
Tumour location (%)	
Upper lateral	42 (42%)
Upper medial	7 (7%)
Lower medial	8 (8%)
Lower lateral	6 (6%)
Central	4 (4%)
Upper central	14 (14%)
Lower central	2 (2%)
Lateral central	11 (11%)
Medial central	6 (6%)
Tumour type (%)	
Invasive lobular	5 (5%)
Invasive carcinoma No Special Type (NST)	67 (67%)
In situ	28 (28%)
Dominant tumour size (mm)	
Invasive, median (IQR)	11 (8–15)
In situ, median (IQR)	11.5 (5–29.5)
Clip post NAC (%)	12 (12%)

Breast density assessed by vision, according to the BI-RADS reporting system (respectively fatty breast tissue, scattered density, heterogeneously dense and extremely dense). Tumour location mentioned per quadrant and midline. Tumour type and dominant tumour size assessed during postoperative pathology examination

*IQR* Interquartile range

### Placement procedure

Fourteen experienced breast radiologists performed 100 RFID procedures (7.1 procedures on average, range 1–25). A 92% success rate was achieved, summarised in Table 2. All radiologists were experienced with RSL, the radiologists from MST also with WL. In 8 procedures (all with ultrasound guidance), the placement failed due to dislocation during retraction of the needle-applicator. Dislocation of the tag occurred in BIRADS density categories A, B, and C. In two patients the distance between tag and lesion (15 mm and 18 mm) were accepted after consultation with the surgeon. Two patients received a second RFID tag, and four patients underwent WL. The median duration of the procedure was five minutes (IQR 3–10 min). The mean VAS pain score of the patient was 1.4 (range 0–6). Median time between tag placement and surgery was 7 days (IQR 4–12 days). 99 RFID tags were placed under ultrasound guidance. One patient received a stereotactic placement due to the dense breast tissue encountered during the initial biopsy procedure. Twelve patients underwent surgery after completing NAC.

**Table 2:** Results of 100 RFID tag placement procedures and 96 RFID-guided breast conserving surgeries

Radiology	Total *n* = 100
Shortest distance marker-tumour on mammography in mm, median (IQR)	0 (0–4.75)
Number of days of RFID tag in situ, median (IQR)	7 (4–12)
Duration of placement procedure in minutes, median (IQR)	5 (3–10)
Number of successful tag placements^a^, *n* (percentage)	92 (92%)
Surgery	Total *n* = 96
Identification rate, n (percentage)	96(100%)
Duration of surgery in minutes, median (IQR)	17 (12–20)
Post-operative wound infection, *n* (percentage)	2 (2.1%)
Pathology	Total *n* = 96
Radical excision rate, *n* (percentage)	89 (92.7%)
Re-excision rate, *n* (percentage)	3 (3.1%)
RFID marker retrieved, *n* (percentage)	96 (100%)
Largest tumour diameter in mm, median (IQR)	12(6.5–16)
Specimen volume(cm^3^) per patient, median (IQR)	45.65 (28–73)
Tumour free margin in mm, median (IQR)	4 (1–6)

*IQR* Interquartile range

^a^Successful placement was defined as 0–5 mm distance between any point of the tag to any point of the tumour

**Fig. 3:**
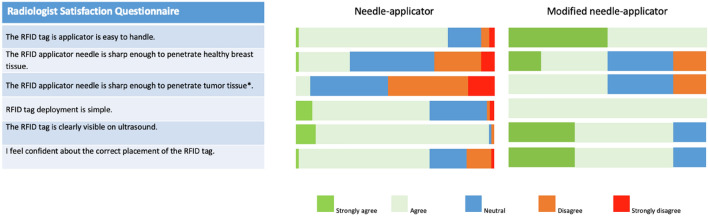
Radiologist satisfaction questionnaire results after the first 50 procedures completed by 14 radiologists (left), and after 50 additional procedures completed by 6 radiologists after needle modification (right). For the first 50 procedures the questionnaire was completed directly after each procedure, whilst for the second 50 procedures the questionnaire was completed at the conclusion of the study. *Based on patients with a solid tumour

**Fig. 4:**
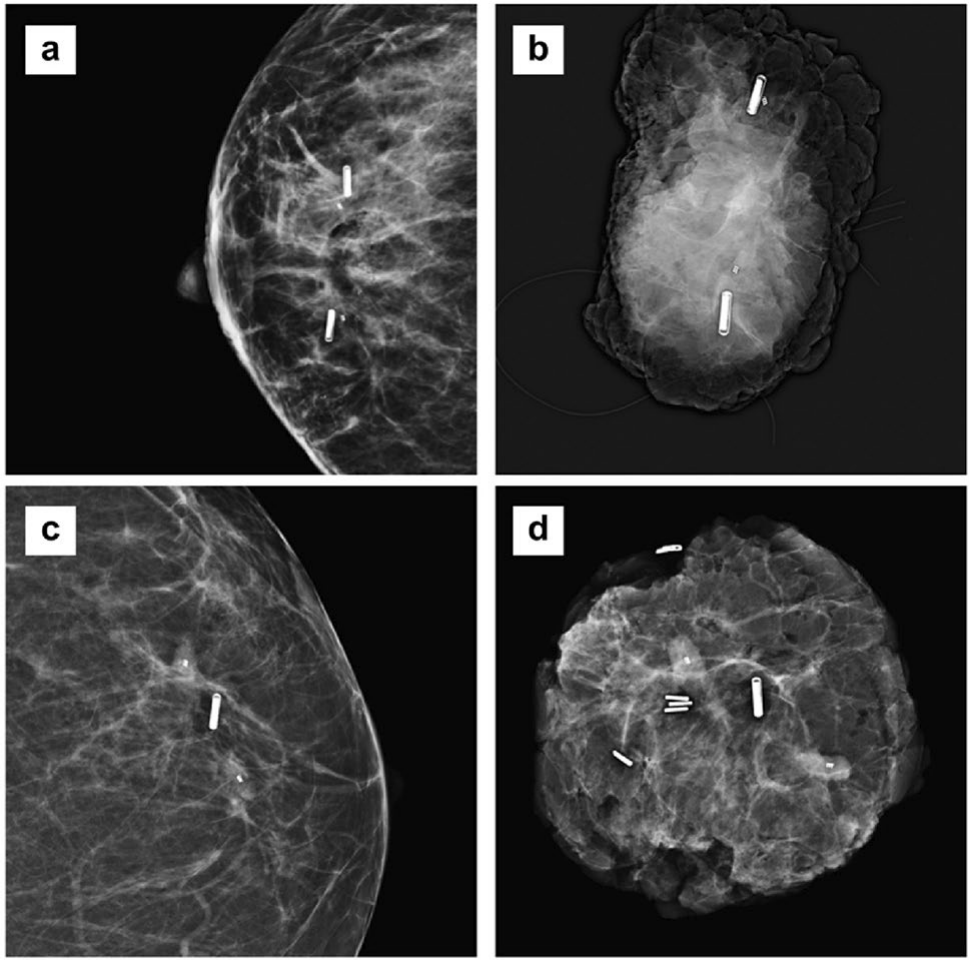
**A** Postplacement mammography (CC-view) of a patient with 2 RFID tags to delineate an area of multifocal breast cancer after NAC; **B** the corresponding specimen radiography (Trident, Hologic) after surgery, with the 2 RFID tag, 2 biopsy clip markers and marking sutures in situ; **C** postplacement mammography (CC-view) of a patient with 1 RFID tag in-between 2 clip markers which both designate an area of microcalcifications; **D** the corresponding specimen radiography (Trident, Hologic) after surgery, with the RFID tag, 2 biopsy clip markers and multiple specimen clips in situ

Experience of the radiologists with respect to the RFID placement is illustrated in Fig. 3 Radiologists were positive about the RFID tag visibility on ultrasound and easy handling of the needle-applicator. However, they experienced difficulty penetrating through dense glandular tissue and solid tumour tissue which led to repeated punctures and manoeuvring of the needle-applicator through the tissue. In relation to this, they assessed the size of the RFID tag and accompanying 12-gauge needle-applicator as large, necessitating local anaesthesia and increasing the risk of bleeding/hematoma. For these reasons, radiologists in one study centre (Diakonessenhuis Utrecht) preferred the radioactive seed procedure over RFID localization leading to premature termination of the study at this site. In response, the needle-applicator was modified to allow for a smooth penetration of dense breast tissue and solid tumours. The modified needle-applicator, implemented after the first 50 procedures, led to an improved user satisfaction (Fig. 3) in the remaining site (MST). In addition, there was an improvement of the successful placement rate from 90% (45 out of 50) to 98% (49 out of 50). However, a substantial learning curve for the radiologists was observed (Fig. 3). Specifically, the radiologists with few or infrequent procedures had a preference towards RSL, whilst radiologists had no preference between RSL/WL and RFID seed after 10 RFID procedures and following frequent placements (monthly). In two patients, two RFID tags were placed to delineate an area of interest. In one patient with multifocal invasive carcinoma an area of 3.2 cm was delineated. In the other case two tags delineated an area of 4 cm DCIS grade 3. In one patient 1 RFID tag was placed between two areas of DCIS grade 3, which were marked with a biopsy clip marker. (Fig. 4).

### Surgical localization

**Fig. 5:**
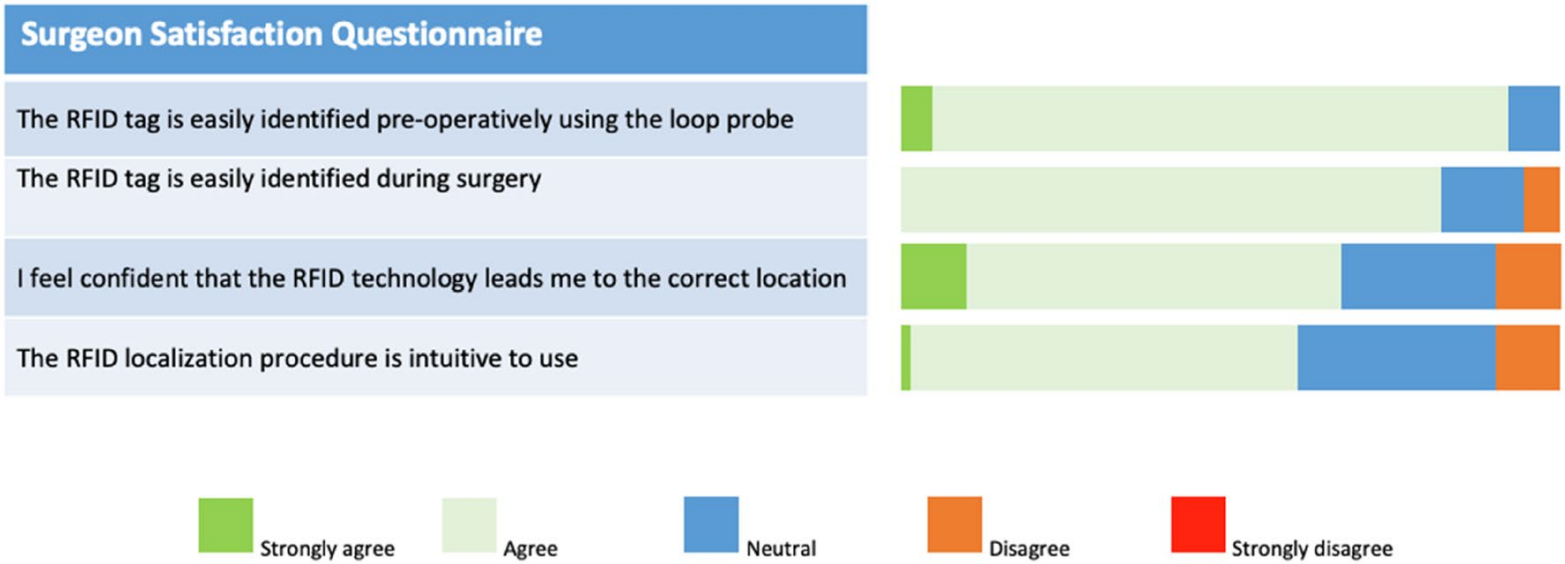
Surgeon satisfaction questionnaire results after the first 50 procedures, completed by 8 surgeons after each procedure

As summarised in Table 2, eight surgeons performed RFID guided BCS in 96 patients (12 procedures on average, range 2–48). Out of the 100 patients included in the surgical evaluation, four individuals experienced dislocation of the RFID tag. In all cases, the dislocation of the RFID tag was detected immediately after its placement and confirmed by the post-placement mammography. Consequently, these patients were excluded from the surgical evaluation exclusively. As per site protocol, these patients received as an alternative method for lesion localization. Transcutaneous identification of the RFID tag with the loop probe was successful in all 96 participants that entered surgical localisation. Median duration of the surgical procedure was 17 min (IQR 12–20 min). All RFID tags were successfully retrieved. User experience with respect to surgery is illustrated in Fig. 5. Surgeons reported that after five to seven procedures, they felt sufficiently familiar with the RFID tag reflecting a signal from two poles (one at each end) instead of a small point source of radioactivity from a radioactive seed.

### Histopathological results

Clear resection margins were obtained in 89 out of 96 patients (92.7%, 95% CI [86.2, 96.7]). Four patients (4.2%) had focally involved resection margins, i.e. 3 patients with DCIS and 1 patient with focal irradical invasive carcinoma (NST).

In three additional patients (3.1%), the resection margins were irradical and these patients required re-excision surgery. Despite a complete radiological response to NAC (per MRI), one patient with infiltrating lobular carcinoma (ILC) had an extensive irradical resection. One patient had irradical resection of invasive carcinoma, i.e. the tumour size was underestimated on preoperative imaging (mammography and ultrasound). The third re-excision was in a patient with extensive florid lobular carcinoma in situ (LCIS) which size was underestimated on preoperative imaging (Mammography and Ultrasound, MRI was not performed). In two additional patients, laceration was observed at multiple sites in the lumpectomy tissue reaching into the tumour, complicating evaluation of the surgical margins (supplementary information).

### Adverse events

Two patients had a postoperative wound infection within 30 days after surgery amongst which one patient after an oncoplastic lumpectomy. Dislocation of the marker during insertion occurred in eight patients (7.7%). After tag insertion, nine patients experienced some bleeding or hematoma after the placement.

At histopathological assessment of the lumpectomy specimens, tissue damage was found in 14 patients (16%). These specimens showed evidence of mild inflammatory and foreign body reaction with fibrosis. In addition, areas of hematomas and large cavities/ruptured tissues were seen in the area where the RFID tag was positioned (supplementary information). The majority of these cases (85%) occurred before implementation of the modified needle-applicator.

## Discussion

This study evaluated the RFID localization procedure in nonpalpable, histologically proven breast cancer including DCIS, and showed clear resection margins in 92.7% of all cases with re-excision indicated in total of 3.1% patients. Importantly, the first 50 procedures indicated difficulties regarding the penetration through dense breast tissue (BIRADS C & D) resulting in tissue damage (hematoma and lacerated tissue) in the area where the RFID tag was positioned. Specifically, multiple lump lacerations were observed in two patients which complicated evaluation of the surgical margins. As these patients did not undergo vacuum assisted biopsy, the tissue damage may be attributed to the RFID procedure. After introduction of the improved needle-applicator there was no tissue damage reported at histopathology. All of these is in line with previous RFID localization studies [9, 10]. Difficulties regarding the penetration led to technical modifications of the needle-applicator (by the manufacturer), i.e. a silicone coating was applied to the needle-applicator. After the modification of the needle-applicator these difficulties were resolved. Stereotactic guidance in one patient with dense breast (BIRADS category C) tissue on mammography (and difficult initial biopsy procedure) was proven successful using modified needle-applicator. Furthermore, supported by recent feasibility studies [10, 18] involving off-label use, it has been identified that the RFID has the potential for targeted excision of suspicious axillary lymph nodes.

The current-standard-of-care in the Netherlands allows for a maximum of 15% irradical excisions [17]. Country-wise statistics in 2020 showed 11.6% re-excision rate for carcinoma in situ, and 4% re-excision rate for invasive carcinoma [17]. This study showed an acceptable percentage of irradical excisions of 7.3%. With respect to 2020, this study showed impressive statistics, i.e. 3.7% re-excision rate for patients with carcinoma in situ, and 2.9% re-excision rate for invasive carcinoma. With respect to other localisation techniques, this study shows similar outcomes regarding resection rates. Specifically, a recent retrospective study by Liang et al. [19] reported negative margins in 91% for WL, 89% for radioactive seed localization, and 89% for magnetic seed. The average lump volume of 45cm^3^ in our study is slightly higher than the findings reported by McGugin et al. [12]. However, it is worth noting that our re-excision rate is significantly lower than in the mentioned article. Specifically, our study observed a re-excision rate of 3.7% for patients with carcinoma in situ and a re-excision rate of 2.9% for patients with invasive carcinoma, compared to a re-excision rate of 17% and 19% reported by McGugin et al. In comparison to our findings, recent research on magnetic seed localization demonstrates a greater lumpectomy volume of 68.5cm3 and higher re-excision rates of 14.4% in invasive carcinoma and 35.3% in DCIS [20].

Clinical challenges for the radiologists (the diameter of needle-applicator and MRI artefact) and surgeons (spatial insight) hampered initial clinical acceptability after the introduction of RFID localization. Therefore, a large non-inferiority trial comparing RFID localization to other localization techniques is necessary to motivate clinical acceptance. Furthermore, additional innovation of RFID is needed for longer-term RFID use in patients undergoing NAC. For the physicians accustomed to RSL (DU) precluded the use of the RIFD in patients receiving NAC, and the benefits of RFID localization did not outweigh the disadvantages of cumbersome localizer placement which resulted in premature termination of the study after nine procedures. However, for the physicians accustomed to WL (Medisch Spectrum Twente), the benefits were worth time and effort to implement RFID localization in clinical care which greatly simplified logistic process. Therefore, clinical acceptability may be related to the standard localization technique used prior to introduction of RFID localization.

In conclusion, RFID technology is a potential alternative for non-radioactive and non-wire localization of nonpalpable breast lesions.

## Supplementary Information

Below is the link to the electronic supplementary material.

Supplementary file1 (DOCX 668 kb)

## Data Availability

The datasets generated during the study will be available from the authors on reasonable request.

## Abbreviations

BCS: Breast conserving surgery
DCIS: Ductal carcinoma in situ
LCIS: Lobular carcinoma in situ
ILC: Infiltrating lobular carcinoma
NST: No special type
WL: Wire localization
RSL: Radioactive seed localization
RFID: Radiofrequency identification
NAC: Neoadjuvant chemotherapy
VAS: Visual Analogue Scale
CC: Craniocaudal
MLO: Mediolateral oblique
MRI: Magnetic resonance imaging
IQR: Interquartile range
CI: Confidence interval
