# Supplementary material for: Radiofrequency localization of nonpalpable breast cancer in a multicentre prospective cohort study: feasibility, clinical acceptability, and safety
Source: Breast Cancer Res Treat. 2023 Jun 15;201(1):67–75. doi: 10.1007/s10549-023-07006-x (PMC10300157; doi:10.1007/s10549-023-07006-x)

**Supplementary information**

Laceration was seen at multiple sites in the tissue reaching into the tumour. A large cystic cavity and haemorrhage was seen in the region where the RFID tag was found.


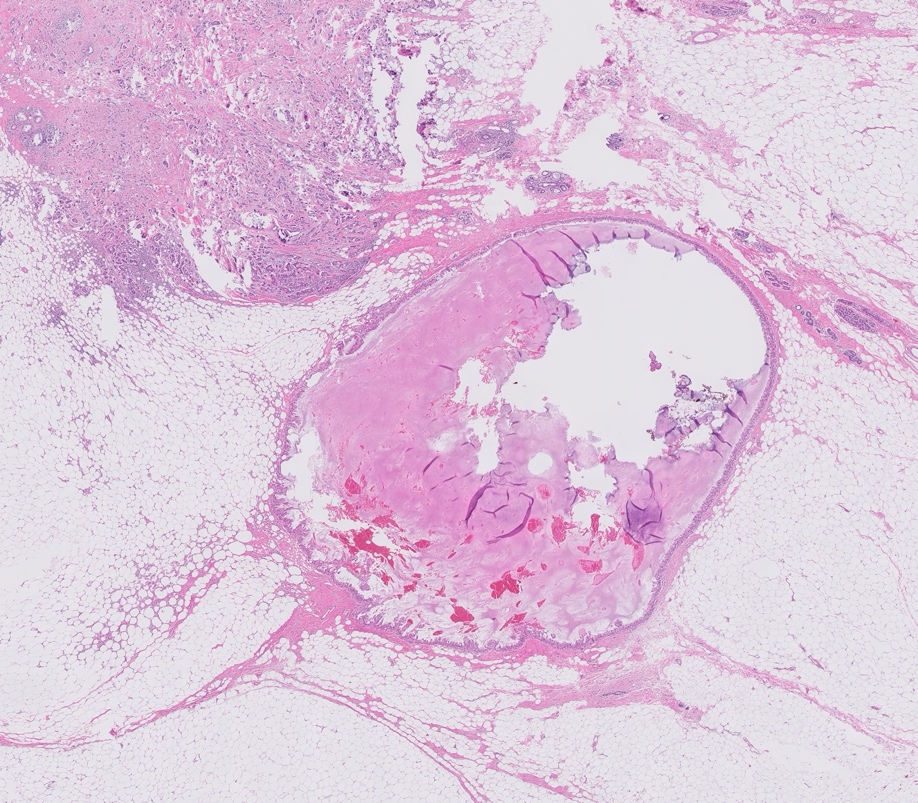


Fibrous irregular mammary parenchyma can be seen around the cavity of the RFID tag. Focal with a cavity of histocytes with fibrosis, fat necrosis and haemorrhage surrounding.


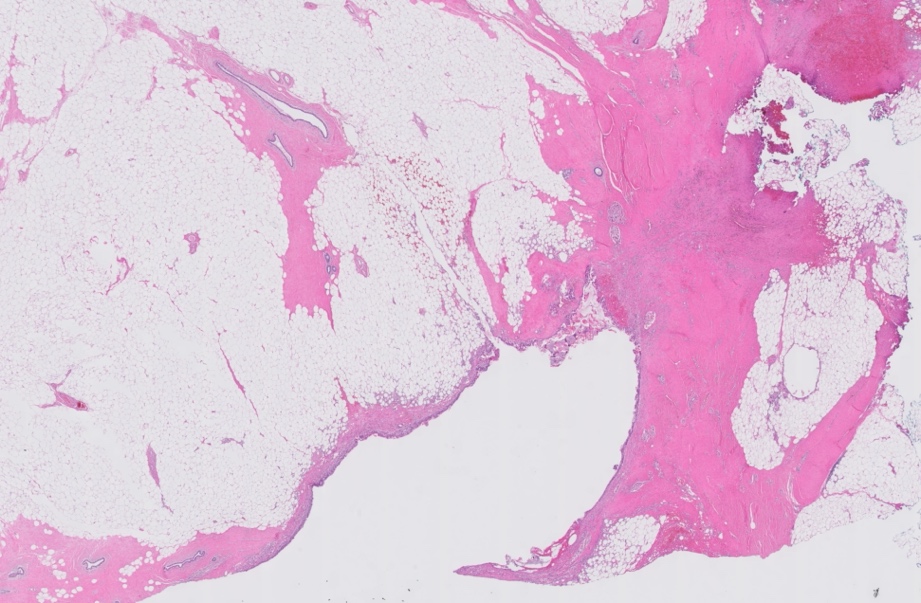

Supplement: Supplementary file 1 — Supplementary file1 (DOCX 668 kb) [file 10549_2023_7006_MOESM1_ESM.docx]
